# In Vitro Genotoxicity of Polystyrene Nanoparticles on the Human Fibroblast Hs27 Cell Line

**DOI:** 10.3390/nano9091299

**Published:** 2019-09-11

**Authors:** Anna Poma, Giulia Vecchiotti, Sabrina Colafarina, Osvaldo Zarivi, Massimo Aloisi, Lorenzo Arrizza, Giuseppe Chichiriccò, Piero Di Carlo

**Affiliations:** 1 Department of Life, Health and Environmental Sciences, University of L’Aquila, I-67100 L’Aquila, Italy; 2 Center for Microscopy, University of L’Aquila, I-67100 L’Aquila, Italy; 3 Department of Psychological, Health & Territorial Sciences, University “G. d’Annunzio” of Chieti-Pescara, I-66100 Chieti, Italy; 4 Center of Excellence on Aging and Translational Medicine—Ce.S.I.-Me.T. University “G. d’Annunzio” of Chieti-Pescara, I-66100 Chieti, Italy

**Keywords:** polystyrene nanoparticles, nanoplastics, genotoxicity, Hs27 human fibroblasts

## Abstract

Several studies have provided information on environmental nanoplastic particles/debris, but the in vitro cyto-genotoxicity is still insufficiently characterized. The aim of this study is to analyze the effects of polystyrene nanoparticles (PNPs) in the Hs27 cell line. The viability of Hs27 cells was determined following exposure at different time windows and PNP concentrations. The genotoxic effects of the PNPs were evaluated by the cytokinesis-block micronucleus (CBMN) assay after exposure to PNPs. We performed ROS analysis on HS27 cells to detect reactive oxygen species at different times and treatments in the presence of PNPs alone and PNPs added to the *Crocus sativus* L. extract. The different parameters of the CBMN test showed DNA damage, resulting in the increased formation of micronuclei and nuclear buds. We noted a greater increase in ROS production in the short treatment times, in contrast, PNPs added to *Crocus sativus* extract showed the ability to reduce ROS production. Finally, the SEM-EDX analysis showed a three-dimensional structure of the PNPs with an elemental composition given by C and O. This work defines PNP toxicity resulting in DNA damage and underlines the emerging problem of polystyrene nanoparticles, which extends transversely from the environment to humans; further studies are needed to clarify the internalization process.

## 1. Introduction

Global plastic production to date is highly related to the environmental pollution by plastic materials [1]. Microplastics (MPs), as fragments <5 mm, but also as fragments with lower dimensions (below 1 mm), are released into the environment [2,3,4].

Once released, MP particles will degrade gradually into nanosized plastics, but at the same time, nanoplastics (NPs, <1000 nm) may be emitted directly into the environment [5]. In recent years, the different aspects of toxicity regarding microplastics have been found in different environmental organisms [5,6,7,8,9,10,11,12]. According to the literature, the upper dimensional limit of nanoplastics goes from a minimum of 100 nm to a maximum of 1000 nm [13,14,15].

Plastics are synthetic or semi-synthetic polymeric materials obtained from natural components such as cellulose, oil, and coal that are used in the most disparate products for their manageability and their rapid production. They are excellent insulators, resistant to corrosion and degradation, which are not optimal for the fate of the environment. Normally produced at a high temperature and by cooling, the individual monomers bind together and form long carbon chains. The most important and used material is polystyrene: an aromatic polymer formed by styrene and petrochemical derivatives including packaging, electronic, and household products.

Plastics as microplastics have also been reported for a long time in the marine environment [2].

The way in which NPs are formed is still largely unknown. The process is sequential: from a macroplastic, one passes through the micro- to then arrive at the nanoplastic. It can be assumed that there is dependence on the plastic material. Polystyrene is the most abundant and reaches concentrations of the order of 10^8^ particles per milliliter [16].

NPs are a subject of study that is still very undervalued and not widespread. The NPs, having reduced dimensions, suffer the impacts of water molecules and suspended ones avoid sedimentation [17]. Being hydrophobic, they can also aggregate with each other based on the pH and the composition of the liquid, for example, polystyrene nanoparticles in the sea aggregate stably by altering their dispersion capacities, mainly in relation to the dimensions. Aggregation, through weak ties, depends on the number and strength of collisions [17]. NPs can also form hetero-aggregation with other materials, which favors their spread [18].

The toxicity, as demonstrated, is not due to the macro components as well as the degradation products of the same, which become more reactive and dimensionally favored to overcome the biological barriers of animals and plants. This damage not only afflicts the ecosystems they live in, but also humans indirectly. First, there is a variation in the trophic chains; second, there is the phenomenon of bioaccumulation: when an animal food reaches the “human table”, it is very likely that it contains not only the plastic directly ingested, but also all that the organisms had consumed, of which the same was fed (biomagnification). The effects of NPs on humans are still mostly unknown, although paradoxically more serious given their small size; thanks to these, they can overcome many biological barriers and enter into circulation, something not possible with the larger dimensions of microplastics. It is important to clarify how these NPs interact with humans and the food chain [18]; two fundamental characteristics of NPs must be taken into consideration: the size/shape and loads, which are the most effective in terms of internalization by cells. NPs of spherical shape are much better absorbed than those of elongated shape and both bind receptor proteins that vary their expression. It is fundamental to investigate such interactions to be able to elaborate a predictive toxicity system. Moreover, the interactions between the surface charges of nanoparticles and the biological membranes are fundamental. Using polystyrene NPs, it was found that these were phagocytosed by macrophages and not internalized by “tissue” cells [18]. Regarding the charge of nanoparticles, the positive NPs are internalized more quickly than the negatively charged ones. [19]. NPs may enter the animal and human food chain [20]. Mussels (*Mytilus edulis*) take NPs and PNPs (polystyrene nanoparticles) in the intestine [21,22] and unicellular green algae adsorb PNPs (<100 nm) [23]. The scallop (*Placopecten magellanicus*) internalizes protein coated polystyrene microparticles [24]. PNPs < 100 nm is present in the marine food chain from algae to fish [25]. PNPs < 500 nm may reach the circulation due to gut absorption in sea urchin embryos [7].

The effects of PNPs (<500 nm) have been tested in vivo in rat [26,27] and in vitro in oral and intestinal models [28]. Very little information is available on the toxicity of PNPs toward human cells and organisms and on the potential risks of adsorbed PNPs [29,30]. Considering the nanosize and the surface exposed of NPs and PNPs, it is urgent to pay more attention to the toxic effects of NPs in environment and human health.

In this work, we focused our attention on polystyrene nanoparticle genotoxicity by considering that NPs induce DNA damage [31,32]. We evaluated the cyto- and genotoxic effects of PNPs on the human fibroblast foreskin Hs27 cell line. The use of human skin fibroblasts as the cell system to test PNP genotoxicity is related to dermo-cosmetics product components enriched by polystyrene microbeads (i.e., scrubs, shampoos, soap, toothpaste and personal care products) [33], which may be fragmented in toxic and genotoxic PNPs and microplastics [34,35].

It will take time for the scientific community to build up the body of hazard and environmental exposure data for a full risk assessment of microplastics and NPs of the types applied in cosmetics and personal care product formulations.

In this context, we investigated the cyto-genotoxic potential of the PNPs after exposing the Hs27 human foreskin fibroblast cell line to different concentrations in the culture medium; following the treatment, we evaluated the viability and metabolic activity of the cells by the MTS assay test of cell proliferation associated with a preliminary screening improved by growth curve. Moreover, we detected reactive oxygen species (ROS) production with PNPs alone and PNPs added with an antioxidant extract of *Crocus sativus* L. stigmas. To estimate the PNP genotoxic potential, we used the cytokinesis-block micronucleus (CBMN) assay. Finally, we carried out PNP morphological analysis through scanning electron microscopy (SEM) equipped with an x-ray microanalysis system to obtain a chemical and semiquantitative characterization of the single elements of the PNPs.

## 2. Materials and Methods

### 2.1. Cell Culture

The in vitro toxicological study was conducted in a cell line, the fibroblast Hs27 (human foreskin, cultures from Public Health England, supplied by Sigma-Aldrich Srl, Milan, Italy).

Cell culture media, trypsin, and all reagents used, unless otherwise indicated, were purchased from Euroclone SpA. The cells were cultured in Dulbecco’s Modified Eagle Medium (DMEM) containing 10% fetal bovine serum, 100 UI/mL Penicillin/Streptomycin, 2 mM L-glutamine, in a HERAEUS incubator (Hera cell 150, Thermo Electron Corporation, Langenselbold, Germany) set with the following parameters: 5% CO_2_ atmosphere, 37 °C temperature. The culture maintenance was carried out under sterile conditions under a biological laminar flow hood. The cells were detached with 0.05% trypsin-0.02% EDTA.

### 2.2. Polystyrene Nanoparticles

The polystyrene nanoparticles (PNPs) were purchased to Sigma Aldrich (catalogue No. 43,302). The particle size was 100 nm, the diameter was 0.100 mm, and the density 1.05 gr/cm^3^. The particles are in aqueous suspension (10% WT).

### 2.3. Saffron: Crocus sativus L. Stigmas Extract

Plant material (*Crocus sativus* L.) was kindly furnished by local farmers in the area of the “Zafferano dell’Aquila PDO” consortium, Navelli, AQ (Italy). Plant extraction (stigmas) was performed according to [36], in our case, however, the extraction was carried out in aqueous solution [37].

### 2.4. Cell–Growth Curve

The cells were seeded at a density of 10,000 cells/cm^2^ in a six multi-well (35 mm in diameter) and when 90% of confluence was achieved, they were counted in a Bürker camera with the dye exclusion Trypan Blue, diluted 1:10. The determination was carried out for 4, 24, and 48 h at different concentrations of 5, 25, and 75 μg/mL of PNPs. In particular, the cells were treated with the different concentrations of PNPs, then readings were taken for each concentration at different times of exposure (4, 24, and 48 h).

Each experimental condition represents a technical triplicate, data refer to the mean and standard error of three independent experiments.

### 2.5. MTS [3-(4,5-dimethylthiazol-2-yl)-5-(3-carboxymethoxyphenyl)-2-(4-sulfophenyl)-2H-tetrazolium] Test

The viability of Hs27 cell line was determined by the MTS assay using a CellTiter Cell Proliferation Test Kit (Promega, Madison, MI, USA). The analysis was performed according to the manufacturer’s protocol. The effect of PNPs (size 100 nm) on cell proliferation was assessed following exposure for 4, 24, and 48 h at different concentrations (5 μg/mL, 25 μg/mL, and 75 μg/mL). Cells were seeded at 5000 cells/cm^2^ and treated after 24 h with different concentrations of PNPs (5 μg/mL, 25 μg/mL, and 75 μg/mL) at the established times in a humidified incubator in a controlled atmosphere (5% CO_2_, 80% humidity, 37 °C). Each experimental condition represents a technical triplicate and data refer to the mean and standard error of three independent experiments. The positive controls (cells treated with 0.1% Triton X-100) were performed with each series of experiments (4, 24, and 48 h). Cell culture absorbance was measured at 490 nm, and cell proliferation was evaluated [38].

### 2.6. ROS (Reactive Oxygen Species) Detection

Cellular ROS concentration was determined according to the “Total ROS Assay Kit 520 nm”. Briefly, 10,000 cells/cm^2^ were seeded in 96-well plates, after 24 h, cells were incubated at 37 °C for 60 min with ROS stain 1X (Thermo Fisher Scientific Inc., Waltham, MA, USA ref. 88-5930) resuspended in dimethyl sulfoxide (DMSO). After incubation, the medium was removed, DMEM was added in the control cells and DMEM containing 5, 25, and 75 μg/mL of PNPs in treated cells. The H_2_O_2_ (150 µM) was added as the positive control. Another plate was performed with the same conditions (5, 25, and 75 μg/mL of PNPs), in which an antioxidant extract of *Crocus sativus* stigmas was added at 25 µg/mL final concentration [37].

Both plates were read at different times in a microplate reader (Perkin-Elmer Victor 3) (λexc 490, λemi535) at T0, T15, T30, T45, T60 min, and T24 h. The fluorescence data T0–T24 h were evaluated for statistical analysis. Each experimental condition represents a technical triplicate, data refer to the mean and standard error.

### 2.7. Cytokinesis-Block Micronucleus (CBMN) Assay

CBMN was carried out with slight modifications according to the protocol of Fenech [39] and OECD guidelines [40]. The Hs27 cell line was seeded in each flask with 2.5 × 10^5^ cells/flask, and after 24 h of culture, the cells were exposed to different concentrations (5 μg/mL, 25 μg/mL, and 75 μg/mL) of PNPs for 48 h. Colchicine was used as a positive control at 5 μg/mL. Cytochalasin B (3 μg/mL) no longer than 24 h after stimulation by PNPs was added to the cell cultures.

Cells were harvested after an additional 24 h and centrifuged for 8 min at 1100 rpm; next, the supernatant was removed, and cells treated for 1 min with 0.075 M KCl hypotonic solution.

Following, the cells were processed and analyzed according to the criteria of Fenech guidelines [39].

Three biological replicates for each sample were used for CBMN analysis with three technical replicates (slides) each.

For each experimental condition, we calculated the cytokinesis block proliferation index (CBPI) to determine the frequency of mononuclear cells, bi- and multinucleated, using the following formula: ((N° mononucleated cells) + (2 × N° binucleated cells) + (3 × N° multinucleated cells))/(total number of cells). Furthermore, for each experimental condition we evaluated the total cells and nuclear buds (NBUDs) as a biomarker of genotoxicity.

### 2.8. Analysis of Polystyrene Particles (PNPs) by Scanning Electron Microscopy SEM

The study of the morphology and the elemental analysis of PNPs were carried out by scanning electron microscopy (Gemini Field Emission SEM 500, ZEISS, Milan, Italy) equipped with an x-ray microanalysis system (EDS Oxford Inca 250 x-act) at the Center of Microscopies, University of L’Aquila.

For PNP characterization, the sample (1 μL) was deposited on a dedicated sample carrier (stub) and then dehydrated in air. Finally, a thin film (5 nm) of chromium was deposited onto the sample using Sputter Quorum 150T ES to make it conductive for measurement purposes.

The SEM observations were carried out at different magnifications, and morphological analysis of the particles was performed simultaneously to obtain the EDS microanalysis of the selected particles.

### 2.9. Statistical Analysis

For the data statistical analysis, we used the Student’s *t* test (unpaired) with post-hoc correction, comparing the value of the treated cells with the respective untreated control, through independent tests. For statistically significant values, * = *p* < 0.05; ** = *p* < 0.005; *** = *p* < 0.0005.

The data were analyzed using the GraphPad Prism software, version 6.0 (© 1995–2015 GraphPad Software, Inc. San Diego, CA 92108). Three independent experiments were performed for all assays applied.

## 3. Results

### 3.1. Cell Growth Curve and MTS

**Figure 1:**
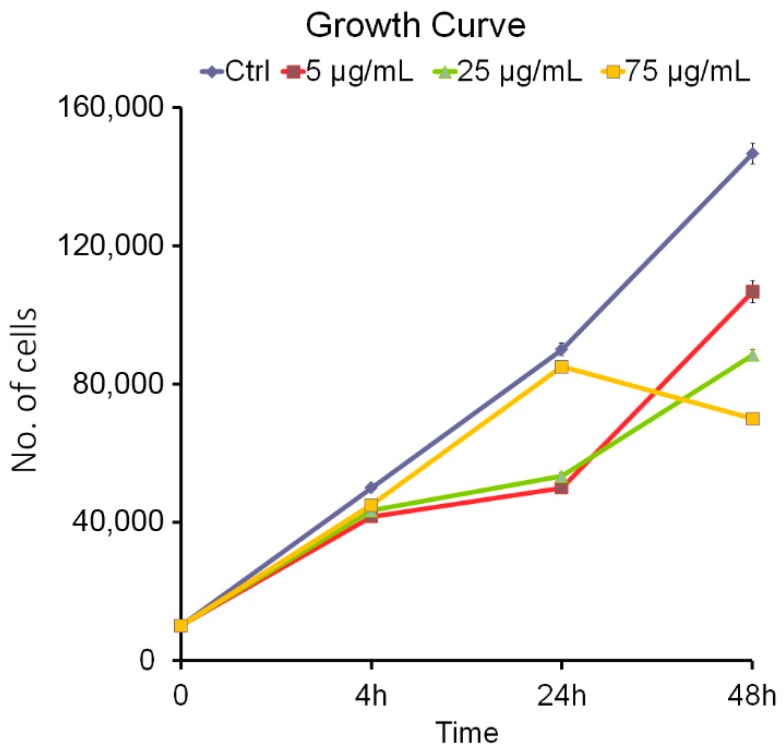
Growth curve in the Hs27 cells was determined by Trypan Blue (counting dye method). The effects of polystyrene nanoparticles (PNPs) exposure were evaluated following exposure at 4, 24, and 48 h at 5, 25, and 75 µg/mL concentrations and compared to the control cells. Error bars represent the standard error of the mean.

Preliminary results are reported in the growth curve for the Hs27 cell line with PNP treatment at different concentrations (Figure 1). In Figure 1, it can be seen that with respect to the control in every experimental condition up to 4 h, there was no significant proliferation decrease, regardless of the concentration. Differences appeared after 24 h, but control and the highest concentration still showed almost identical results. The exposure of the cells to 75 μg/mL of PNPs at 48 h showed a sudden decrease. The trend of the growth curve was due to the possible tendency of the nanoparticles to aggregate (PNPs in this work as in general the nanoparticles do [30]), this explains why at the lower concentrations there were no statistically significant results; on the contrary, at the concentration of 75 μg/mL and at a longer time of incubation (48 h), we can assume that PNP particles aggregates tend to enhance and interact with cell proliferation.

The cytotoxicity of the PNPs was measured by the MTS cell viability test, which evaluates the metabolic activation of Hs27 cells after treatment at different concentrations.

**Figure 2:**
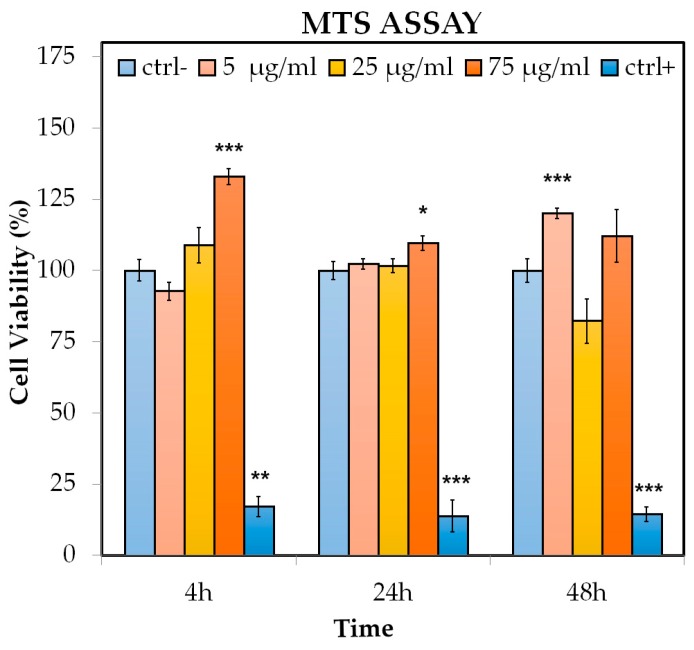
MTS [3-(4,5-dimethylthiazol-2-yl)-5-(3-carboxymethoxyphenyl)-2-(4-sulfophenyl)-2H-tetrazolium] test in Hs27 cells: the effects of polystyrene nanoparticles (PNPs) on cell proliferation were evaluated following exposures at 4, 24, and 48 h at 5, 25, and 75 µg/mL concentrations and compared to the control cells. Triton-X-100 0.1% was used as a positive control. Significance values * = *p* < 0.05; ** = *p* < 0.005; *** = *p* < 0.0005; error bars represent the standard error of the mean.

The test determines whether cells increase their metabolic activity, measuring the reduction of MTS by a formazan soluble in the culture medium, as MTS reduction only occurs in viable and metabolically active cells. Compared to the control cells, the data at 4 h showed a significant viability increase at 75 μg/mL (about 33%). After 24 h, again, the treatment at 75 μg/mL of PNPs was statistically significant; finally at 48 h, the results showed an increase only at 5 μg/mL PNPs, which was about 20%. Triton-X-100 0.1% was used as a positive control and induced a significant decrease in viability after 4, 24, and 48 h treatment in PNPs (Figure 2). We speculate that the observed viability trend is not dose dependent and that there is no significant variation in cell viability.

### 3.2. Tests of Micronuclei with Block of Cytokinesis with Cytochalasin B “CBMN Assay”

From the data obtained from the micronucleus test, we calculated the CBPI index “Cytokinesis Block Proliferation Index” to evaluate the cellular proliferation progression and therefore the cytostatic and cytotoxic effects, followed by the different concentrations of PNPs (5, 25, and 75 μg/mL).

**Figure 3:**
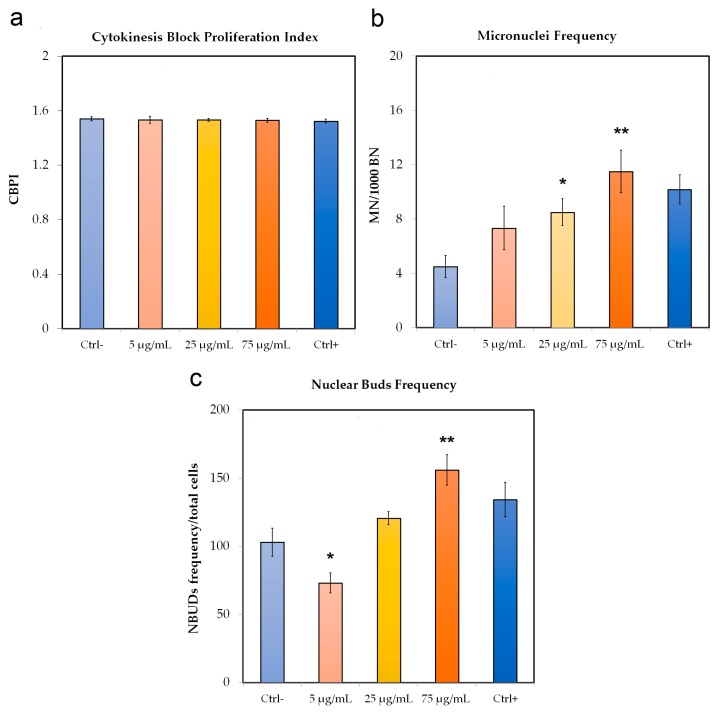
Micronuclei (BNMN), CBPI, and Nuclear Bud (NBUDs) expression in the Hs27 cells treated with polystyrene nanoparticles (PNPs): CBPI, “Cytokinesis Block Proliferation Index” (**a**), Micronuclei (**b**) and Nuclear Buds (**c**) were evaluated at 5, 25, and 75 μg/mL concentrations after exposure to PNPs. CBPI = ((N° mononucleated cells) + (2 × N° binucleated cells) + (3 × N° multinucleated cells))/(total cell number). The number of micronuclei refers to 1000 binucleated cells. The number of Nuclear Buds refers to a total of 1000 binucleated cells. Colchicine (5 μg/mL) was used as a positive control. Significance values * = *p* < 0.05; ** = *p* < 0.005; *** = *p* < 0.0005; error bars represent the standard error of the mean.

**Figure 4:**
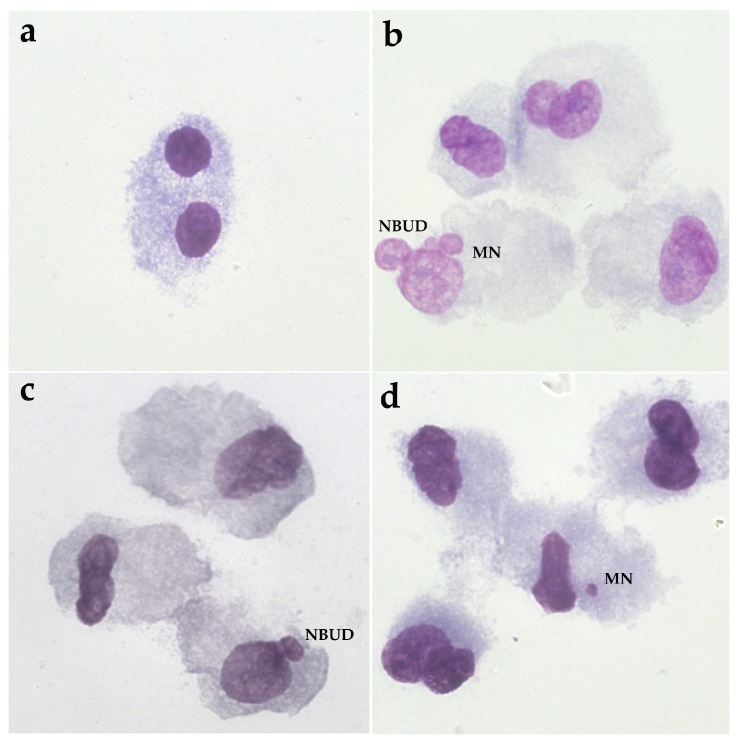
CBMN assay in the Hs27 cells: DNA damage after PNP treatment. (**a**) Binucleated cell, (**b**) cell with micronuclei and nuclear bud, (**c**) cell with nuclear bud, (**d**) cell with micronuclei. Magnification 100×.

Compared to the control condition, the CBPI obtained from the cells incubated with the PNPs was, in every condition data, not statistically significant (Figure 3a). Regarding the induction of micronuclei (BNMN), we observed a significant increase dose-dependent at 25 μg/mL and 75 μg/mL where we observed an increase of about 38% and 52%, respectively (Figure 3b). Furthermore, we analyzed the presence of NBUDs (Figure 3c), which originate from the nucleus as extroflections of nucleoplasmic material or as micronuclei connected to the nucleus by a bridge [40]. Our result shows a significant decrease at 5 μg/mL (about 30%) with respect to the control, and on the contrary, an increase at 75 μg/mL of about 50%. In Figure 4, we can see DNA damage as micronuclei and NBUDs in Hs27 cells after PNP treatment.

### 3.3. ROS Detection

**Figure 5:**
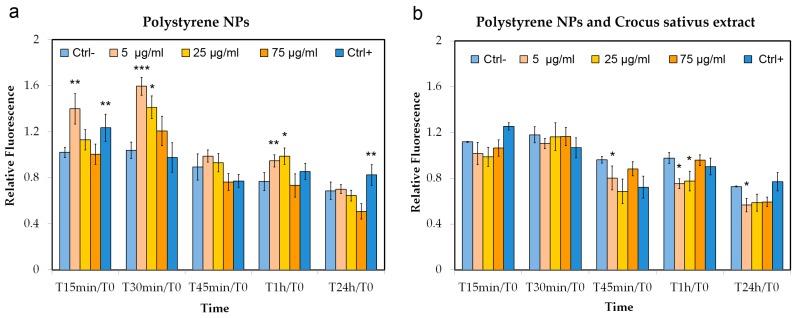
ROS detection in Hs27 cells: the effects of polystyrene nanoparticles (PNPs) on ROS production were evaluated following exposure at 0, 15, 30, 45, 60 min, and after 24 h at 5, 25, and 75 µg/mL concentrations. Each time refers to T0. Hs27 ROS production with PNPs (**a**) and Hs27 ROS production added with antioxidant stigmas extract of *Crocus sativus* (25 µg/mL) (**b**). H_2_O_2_ (150 μM) was used as a positive control. Significance values * = *p* < 0.05; ** = *p* < 0.005; *** = *p* < 0.0005; error bars represent the standard error of the mean.

Time course experiments were performed to comparatively evaluate the possible ROS production in Hs27 cells at different concentrations of PNPs (5, 25, and 75 µg/mL) and PNPs added with an antioxidant extract of *Crocus sativus* stigmas (25 µg/mL). Figure 5a shows that treatment with 5 μg/mL NPs induces highly significant stimulation of ROS production in the cell line, starting from T15 min/T0 of treatment. At T30 min/T0, we statistically increased in ROS production at 5 μg/mL (*p* < 0.0005) and 25 μg/mL (*p* < 0.05) concentrations. The increase ROS level at T1 h/T0 was still statistically significant with respect to the control at 5 μg/mL (*p* < 0.005) and 25 μg/mL (*p* < 0.05) concentrations, but we also noticed that the same concentrations with respect to T30 min/T0 had a strong decrease in ROS production. At T24 h/T0, there are no significant variations in ROS production with respect to the control cells. In Figure 5b, we reported the level of ROS production with PNPs added together with saffron extract: we observed that in the presence of the extract, the ROS production was lower. Significant data obtained by comparing the PNP treatment and PNPs added with saffron are reported in Table 1, and it is noticeable that there was a significant decrease in reactive oxygen species at 5 μg/mL and T15 min/T0, T30 min/T0, and T1 h/T0. In particular at T30 min/T0, we had a ROS decrease by about 30%. Regarding the 25 μg/mL concentration, the results were statistically significant at T30 min/T0 and T1 h/T0 and a ROS reduction of about 18% and 22%, respectively.

### 3.4. Analysis of PNPs by SEM Scanning Electron Microscopy

**Figure 6:**
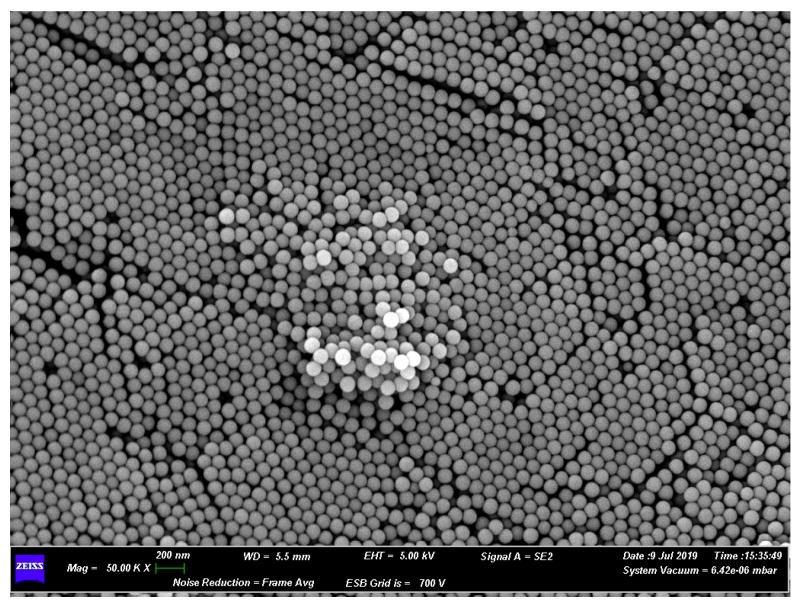
Scanning electron microscopy (SEM): morphological analysis of polystyrene nanoparticles (PNPs) with scanning electron microscopy.

PNPs information were obtained by evaluating their morphology and elemental composition. With electron microscopy, we undertook a morphological analysis through different images to test the particle size (Figure 6). Regarding the composition, an investigation was made with EDS microanalysis to assess whether there were impurities such as heavy metals that could affect the experiment. During the analysis of the sample, properly treated, we noticed that polystyrene nanospheres tended to form a reticular structure thanks to their homogeneous shape. Furthermore, it could be seen that the average particle size was around 100 nm, according to the manufacturer’s specifications (Figure 6).

**Figure 7:**
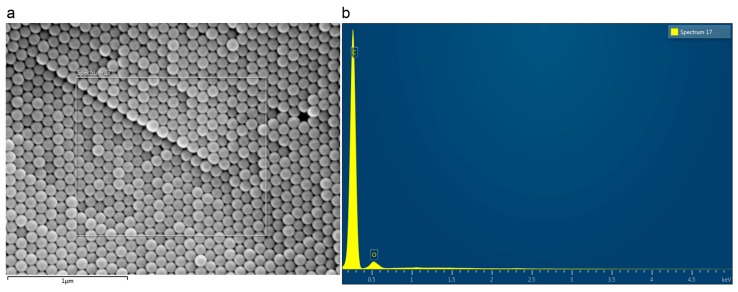
Energy dispersive x-ray analysis (EDX spectroscopy) analysis of polystyrene nanoparticles (PNPs) to characterize elemental composition, area of sample (**a**) and relative elemental spectrum (**b**).

Taking advantage of EDX spectroscopy (energy dispersive x-ray analysis), we evaluated the elemental composition of the sample (Figure 7). The technique provides information on the elemental composition, hence the spectrum only shows the presence of the carbon elements, reinforcing the idea that the only component was polystyrene and oxygen due to the presence of the water residue.

## 4. Discussion

Global plastic production increases annually [41], with an estimated 4.8 to 12.7 million metric tons of plastic entering the oceans each year [42], posing a threat to seabirds [43], fish [44], turtles [45], and marine mammals [46]. Dispersed plastic is a new emergency for environmental health, and the greatest danger is derived from the products of their degradation. NPs are dispersed in the soil, air, and water; in particular PNPs are the most subjected to degradation. Some new evidence of the toxic potential of PNPs has emerged from the present study, particularly with regard to the genotoxicity.

Tests of the viability of MTS cells by evaluating the metabolic activity of Hs 27 cells exclude an inhibitory action of PNPs on metabolic activity; this activity increased after PNP treatment most likely as a response to cellular stress. This hypothesis is supported by the results of the CBMN tests regarding the treatments with the lowest concentrations of NPs. Genotoxic damage was observed at concentrations above 5 µg/mL, which produced results comparable to MTS tests. Thus, high concentrations of PNPs seem to be necessary to produce appreciable cell damage in relation to exposure times. These in vitro data are quite indicative of the genotoxicity of PNPs and provide indirect evidence of the ability of PNPs to penetrate cells, as widely reported for other particles of similar size to NPs [30] and PNPs [47].

From our results, it is clear that by analyzing the metabolic activity in relation to the production of ROS, treatment with PNPs is able to determine oxidative stress inside the cells. In agreement with the literature [30], the result obtained by us show a high production of ROS within the first 30 min and a decrease afterward, due to the detoxification systems that the cell puts in place. Moreover, we observed that the ROS production decreased when PNPs were added together with the saffron extract. Hence, the free radical scavenging ability of saffron [48] is also expressed in human fibroblasts in which oxidative stress is produced by PNPs. This ability is related to the phenolic/flavonoid contents of saffron *Crocus sativus* L. stigmas known to play a role in preventing oxidative damage caused by free radicals and inhibiting hydrolytic and oxidative enzymes [49]. Thus, the Hs27 human fibroblasts exposed to PNPs suffer damage both in terms of genotoxicity and oxidative stress and the antioxidant power of saffron extract may be able to contrast the ROS formation.

According to the SEM-EDX analysis, PNPs are composed exclusively of C and O, and therefore the physical–chemical properties, and consequently the toxic effects are attributable to the size, shape, surface properties, reactivity, and solubility, all characteristics that influence the ability to induce damage within the cells [50]. The more that particles reach a nanosize, the more their surface area exposed with reactive chemical groups extends.

Our current approach to study the toxicological potential of PNPs raises some important points such as to determine how the particles are internalized by the cells; particles with dimensions of about 100–200 nm are internalized through endocytosis mechanisms, in contrast, larger ones are absorbed through phagocytosis [30,51]. The available information on the toxicity of PNPs in vivo is poor [30,52,53]. In this regard, considering environmental pollution, adverse factors could be invoked for NPs and PNPs such as the risk that they can adsorb, concentrate, and release environmental pollutants into the organisms, thus acting as transporters [54,55,56].

## Figures and Tables

**Table 1:** ROS detection comparison in Hs27 cells treated with polystyrene nanoparticles (PNPs) and PNPs added with the *Crocus sativus* stigmas extract. Values are means ± SD.

Time Ratio	5 µg/mL		*P* Value	25 µg/mL		*P* Value
	PNPs	PNPs with *Crocus s.*		PNPs	PNPs with *Crocus s.*	
T15 min/T0	1.4 ± 0.004	1.01 ± 0.004	<0.05	1.13 ± 0.005	0.99 ± 0.002	
T30 min/T0	1.60 ± 1.2 × 10^−5^	1.10 ± 0.001	<0.0005	1.41 ± 0.007	1.16 ± 0.001	<0.05
T1 h/T0	0.95 ± 0.0013	0.75 ± 0.001	<0.005	0.99 ± 0.007	0.78 ± 0.002	<0.05
